# Thermodynamic picture of ultrafast charge transport in graphene

**DOI:** 10.1038/ncomms8655

**Published:** 2015-07-16

**Authors:** Zoltán Mics, Klaas-Jan Tielrooij, Khaled Parvez, Søren A. Jensen, Ivan Ivanov, Xinliang Feng, Klaus Müllen, Mischa Bonn, Dmitry Turchinovich

**Affiliations:** 1 Max Planck Institute for Polymer Research, Ackermannweg 10, Mainz 55128, Germany; 2 ICFO—Institut de Ciències Fotòniques, Mediterranean Technology Park, Castelldefels, Barcelona 08860, Spain

## Abstract

The outstanding charge transport properties of graphene enable numerous electronic applications of this remarkable material, many of which are expected to operate at ultrahigh speeds. In the regime of ultrafast, sub-picosecond electric fields, however, the very high conduction properties of graphene are not necessarily preserved, with the physical picture explaining this behaviour remaining unclear. Here we show that in graphene, the charge transport on an ultrafast timescale is determined by a simple thermodynamic balance maintained within the graphene electronic system acting as a thermalized electron gas. The energy of ultrafast electric fields applied to graphene is converted into the thermal energy of its entire charge carrier population, near-instantaneously raising the electronic temperature. The dynamic interplay between heating and cooling of the electron gas ultimately defines the ultrafast conductivity of graphene, which in a highly nonlinear manner depends on the dynamics and the strength of the applied electric fields.

## Abstract (web summary)

A linear energy–momentum relation of graphene results in a high direct-current electron mobility, but this is not necessarily true at terahertz frequencies. Here, the authors show that its ultrafast conductivity is dependent on a highly nonlinear interplay between heating and cooling of the electron gas.

## Graphical abstract

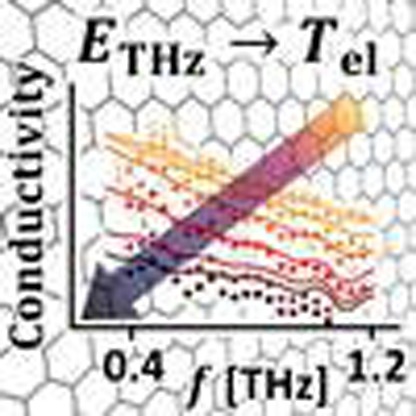

Graphene stands out among other electronic materials for its unique band structure, which in the broad vicinity of the Dirac point (the point of lowest electronic energy) is characterized by a linear relation between the energy *E* and momentum *p* of an electron *E*=*pv*_F_ , where *v*_F_≈*c*/300 is the Fermi velocity—the electron group velocity, and *c* is the speed of light in vacuum^1^. The linear energy-momentum dispersion implies the massless, photon-like behaviour of charge carriers, leading to extraordinary electronic properties of graphene, such as very high direct-current carrier mobility^1^,^2^,^3^,^4^, and enabling a multitude of novel applications of graphene in electronics and opto-electronics^5^,^6^,^7^,^8^,^9^,^10^.

The very high direct-current conductivity in graphene^1^,^11^,^12^ is well-understood within the traditional concept of electrical conduction by the Fermi-edge electrons^3^,^4^, also applicable to metals. At the same time, the exceptional conductive properties of graphene are not necessarily maintained in the regime where the applied electric field oscillates at very high, THz rates corresponding to picosecond or sub-picosecond duration of field cycles. Many electronic applications of graphene are expected to operate in this ultra-high-speed regime, such as THz emitters^13^,^14^ and detectors^15^,^16^, ultrafast transistors^9^,^10^, plasmonic devices^17^,^18^, modulators^19^ and so on. Yet a clear physical picture of graphene conduction in this technologically important ultrafast regime, especially under strong THz fields, has not yet been established^20^,^21^,^22^,^23^.

In the following, we present a unifying view on electron transport in graphene in arbitrary ultrafast electric fields. We demonstrate that the conductivity of graphene in a very wide range of applied electric field strengths and frequencies can be described within a simple thermodynamic approach: by taking into account the statistically determined thermal balance maintained within the entire electron population of graphene interacting with the applied electric field, and without requiring detailed knowledge of the microscopic electron kinetics.

## Results

### Experiment and sample

**Figure 1:**
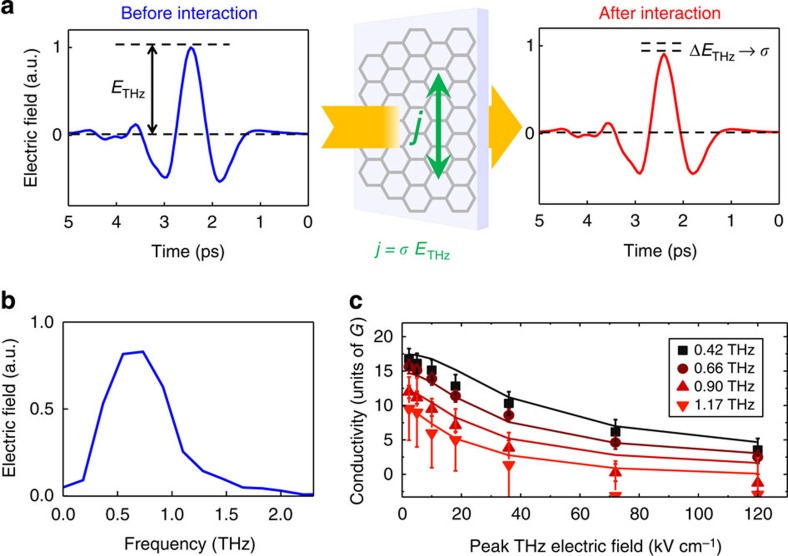
Probing ultrafast charge transport in graphene. (**a**) An oscillating THz pulse with controllable peak field *E*_THz_ incident from free space induces a time-dependent current in graphene *j*=*σE*_THz_, where *σ* is graphene conductivity, which leads to the attenuation of the THz field *ΔE*_THz_. The Fourier transforms of the THz pulses measured before and after the interaction with graphene yield its frequency-dependent conductivity. Thus, the conductivity of graphene is determined for all the frequencies contained in the single-cycle THz pulse, whose amplitude spectrum is shown in **b**. The experiment in **a** is repeated for several values of the THz peak field strength in the range 2.3–120 kV cm^−1^, yielding the ultrafast and contact-free characterization of graphene conductivity over a wide range of applied field strengths and frequencies. Shown in **c**, our measurements demonstrate a significant reduction of graphene conductivity with increasing electric field frequency and strength. Symbols—measured real-valued conductivity of graphene as a function of THz field strength and frequency. Lines—calculation based on the time-dependent thermodynamic balance in electron population, as explained in the text. The conductivity is expressed in quantum units of *G*=*e*^2^/4*ħ*=6.04 × 10^−5^ S (*e* is elementary charge, and *ħ=h*/2*π* is the reduced Planck's constant). The error bars are the s.d. in the measurements.

To study ultrafast charge transport in graphene, we use an all-optical, contact-free conductivity sampling method called nonlinear ultrafast THz spectroscopy^24^,^25^,^26^ (see Supplementary Fig. 1 and Supplementary Methods for details). In short, a single-cycle pulse of THz electromagnetic radiation with controllable peak electric field strength *E*_THz_ propagates through the sample and induces a current *j*=*σE*_THz_ in graphene, proportional to its conductivity *σ*, as shown in Fig. 1a. The single-cycle nature of the THz pulses ensures their large spectral bandwidth, as shown in Fig. 1b. By analysing the THz pulse transmission through the sample, its conductivity is determined over a wide parameter space of peak field strengths (2.3–120 kV cm^−1^) and frequencies (0.4–1.2 THz)^27^ (see Supplementary Methods for details on the data analysis). The contact-free, all-optical nature of the THz conductivity measurement directly provides the intrinsic response of the material, excluding any influence of the measurement circuitry typical for traditional electrical characterization methods.

Our single-layer graphene sample is grown by chemical vapour deposition (CVD) on copper and deposited on a silica substrate^12^,^28^ (see Supplementary Methods for details on sample growth). Initial sample characterization using Raman spectroscopy (Supplementary Fig. 2) and linear THz spectroscopy (Supplementary Fig. 3) yielded the Fermi energy (chemical potential at room temperature) of *E*_F_=0.07±0.01 eV, corresponding to a sheet carrier density of *N*_s_=(6.0±2.0) × 10^11^ cm^−2^, and the electron momentum scattering time at the Fermi level of *τ*(*E*_F_)=140±20 fs. All measurements in this work were performed at room temperature.

An overview of the experimental results is shown in Fig. 1c. We find, similar to previous reports^21^,^22^,^23^, that the overall transmission of the THz field through graphene increases with increasing peak THz field strength. However, we also find a significant frequency dependence of the graphene conductivity in strong fields. Our measurements show a dramatic reduction of graphene conductivity with increasing THz frequency—that is, the faster the electric field oscillates the more resistive the graphene becomes, for any strength of applied electric field, as shown in Fig. 1c. While the conductivity of graphene is still high at low frequency and weak electric fields, in the regime of ∼1 THz, ∼100 kV cm^−1^ fields the conductivity almost vanishes. As explained below, this dramatic conductivity reduction is a direct result of the accumulation of thermal energy, absorbed from the applied ultrafast electric fields, within the highly correlated electronic system of graphene, which on the THz timescale behaves as a hot electron gas. This affects the conductivity of graphene, which is dependent on the electronic temperature.

### Terahertz conductivity of thermalized electron gas in graphene

**Figure 2:**
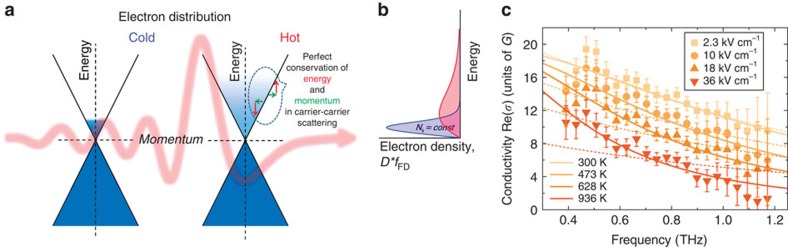
Essentials of the thermodynamic effect on ultrafast transport in graphene. (**a**) Cold and hot-carrier distribution in graphene, before and after interaction with the strong-field THz signal, respectively. The illustration of perfect energy and momentum conservation in electron–electron scattering, resulting in sub-50 fs thermalization of the electron gas in graphene. (**b**) Energy dependence and conservation of the free carrier density for the cold and hot-carrier distributions illustrated in **a**. The elevated electron temperature leads to a smeared-out carrier distribution, and accordingly lowered chemical potential. (**c**) Frequency-dependent conductivity spectra of graphene, measured at selected peak THz field strengths in the range of 2.3–36 kV cm^−1^ (symbols). The data is well-described by the thermodynamic model equation (1) (solid lines) assuming long-range Coulomb impurity electron momentum scattering. The corresponding effective carrier temperatures are indicated in the figure. The dashed lines correspond to the calculation for the short-range disorder momentum scattering scenario. The error bars are the s.d. in the measurements.

A large ensemble of electrons, an electron gas, has a natural, statistically determined state—the thermalized state. In such a state, all electrons in the gas are equilibrated and are characterized by a common temperature *T*_el_, while the total kinetic energy of all electrons makes up the thermal energy of the entire gas—the electron heat. The energy distribution of the electrons in a thermalized gas is described by the Fermi–Dirac function 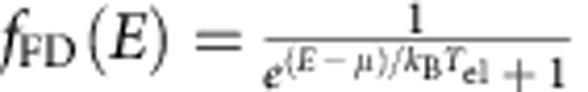, where *μ* is the chemical potential and *k*_B_ is Boltzmann's constant. The process of thermalization, the equipartition of energy among all electrons in a gas, occurs via electron–electron interactions—elastic scattering processes in which the electrons exchange their energy and momentum. In graphene, this process is extremely efficient^29^, which is facilitated by its unique linear energy-momentum dispersion *E(p)*=*pv*_F_. Indeed, given the available phase space, any two electrons in graphene *i* and *j*, occupying any two states (*E*_*i.j*_*, p*_*i,j*_), can change their states, so that if one electron increases its energy and momentum by *ΔE* and *Δp*, respectively, the second one will decrease its energy and momentum by the exact same amounts *ΔE* and *Δp*, as illustrated in Fig. 2a. As a result of such perfect energy and momentum conservation in electron–electron energy exchange events, the electron–electron interaction in graphene is extremely fast. Indeed, it was found that the thermalization of the electron population in graphene occurs on a sub-50 fs timescale^30^,^31^,^32^,^33^,^34^,^35^,^36^. This ultrafast thermalization, much faster than the ∼1 ps timescale of THz field oscillation (see Fig. 1a), allows one to consider the electron population in graphene as thermalized at all times during the interaction with the THz fields. Accordingly, the process of adding extra energy to the electronic system in graphene can be treated as a thermodynamic effect of adding more heat to the electron gas, simply leading to an increase in the electron temperature *T*_el_. This motivates our thermodynamic, rather than microscopic, approach to describe the THz conductivity in graphene: instead of following the microscopic electron kinetics, on the THz timescale we can consider the macroscopic statistical properties of the whole ensemble of electrons in graphene.

Examining the thermal distribution (Fermi–Dirac) function, we note that, unlike in metals which typically have a Fermi energy on the order of several electron volts, in doped graphene with a typical *E*_F_∼100 meV the thermal broadening of the electron distribution can easily be of the same order as the Fermi energy itself, especially at elevated temperatures (>1,000 K). That is, for the description of the electric conductivity in graphene one should consider not only the electrons at the Fermi level (as is the case for metals with a ‘sharp' Fermi edge), but a broader electron distribution, as illustrated in Fig. 2b. With this in mind, we describe the frequency-dependent intra-band conductivity of a thermalized electron gas in graphene *σ*(*ω*) using^3^,^4^,^37^


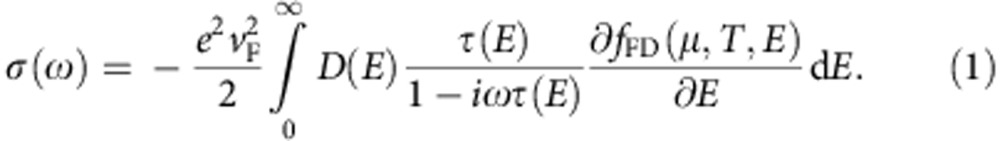


Here 
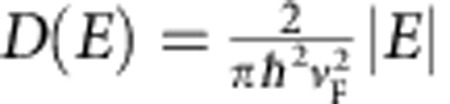
 is the energy-dependent density of states in graphene^3^, and *τ*(*E*) is the electron momentum scattering time, which may also be energy dependent.

In Fig. 2c, as symbols, we show the THz conductivity spectra of graphene measured in the lower range of applied THz field strengths in our experiment, 2.3–36 kV cm^−1^. We first consider the case of the weakest applied THz field of 2.3 kV cm^−1^, where the amount of energy transferred to the electron system of graphene is negligible, and find that equation (1) perfectly describes the result of the measurement with the material parameters independently obtained from the initial sample characterization: *T*_el_=300 K, *μ*=*E*_F_=0.07 eV and *τ*(*E*_F_)=140 fs (see Fig. 2c).

Increasing the THz field strength leads to a significant reduction of the graphene conductivity, especially at higher frequencies. As applying stronger fields to graphene facilitates more energy transfer to its electronic system, this leads to a modification of the electron distribution *D*(*E*)*f*_FD_(*E*), which now corresponds to a certain elevated electron temperature *T*_el_, as illustrated in Fig. 2a,b. The higher electron temperature leads to a smearing of the Fermi–Dirac distribution. Importantly, in graphene with its electronic density of states being directly proportional to energy 
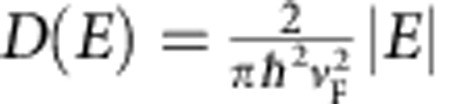
, the increase in electron temperature is accompanied by a lowering of the chemical potential *μ*. The reason for this is that the incident THz field only induces intra-band transitions, and hence the carrier density must remain conserved at all times: 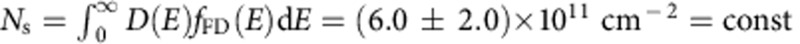, as illustrated in Fig. 2b. If the chemical potential *μ* remained constant, an increase in electron temperature would lead to an increase in carrier density. Therefore, to provide for the necessary carrier density conservation *N*_s_=const, the increase in electron temperature must necessarily be accompanied by a corresponding decrease in chemical potential. This provides a qualitative explanation of the observed reduction of the intra-band THz conductivity for a hotter electron distribution in graphene, shown in Fig. 2c, which is based on the argument of conservation of total spectral weight^38^,^39^: the lower chemical potential at higher electron temperatures implies that inter-band, infrared transitions at lower energies (that were Pauli-blocked before) are now allowed. This leads to a larger spectral weight for the inter-band conductivity and therefore to a decrease of the spectral weight for the intra-band, THz conductivity, as we indeed observe.

### From electronic heat to terahertz conductivity

For a quantitative description of the measured THz conductivity spectra shown in Fig. 2c, we directly apply equation (1) within the constraint of carrier density conservation *N*_s_=const (yielding the chemical potential *μ* at any given temperature as explained above), and use the electronic temperature *T*_el_ as a free fit parameter. At this point, not only the temperature-dependent electron distribution (see Fig. 2b), but also the energy dependence of the momentum scattering time *τ*(*E*) should be considered. To simplify our model, we consider two main electron momentum scattering regimes in graphene (see ref. 4 and references therein), both resulting in an energy-dependent *τ*(*E*): (i) long-range scattering on Coulomb impurities, and (ii) short-range scattering on disorder. The former is expected to dominate in CVD graphene, such as our sample, with the momentum scattering time being directly proportional to the electron energy *τ*(*E*)=*γE* (ref. 40). The latter leads to a momentum scattering time inversely proportional to the electron energy *τ*(*E*)=*β*/*E* (ref. 4). Here *γ* and *β* are the proportionality constants, which can be readily established experimentally from the initial sample characterization at *T*_el_=300 K, as *γ*=*τ*(*E*_F_)/*E*_F_ and *β*=*τ*(*E*_F_) × *E*_F_.

In Fig. 2c, we show the results of applying equation (1) to the data using both momentum scattering scenarios: long-range Coulomb scattering (solid lines), and short-range disorder scattering (dashed lines). The measured data in the whole peak THz field range 2.3–36 kV cm^−1^ are indeed best described using the scenario of long-range Coulomb scattering, as expected for CVD graphene. We find effective electron temperatures in the range 300–936 K. At the same time, the alternative scattering scenario does not describe our data very accurately. Nevertheless, qualitatively the result of both electron momentum scattering regimes in graphene is the same: they both lead to lower electronic conductivity with increasing frequency and strength of applied electric fields, as a result of adding heat to the electron gas in graphene. The validity of this thermodynamic model of conductivity, assuming the electron momentum scattering on Coulomb impurities, is further confirmed by using it on a different CVD graphene sample with a different set of material parameters, mainly a higher Fermi energy at 300 K (see Supplementary Fig. 4).

In the very basic approach shown in Fig. 2c, we directly applied equation (1) using the electron temperature as an effective free parameter, without considering the temporal dynamics of electron heating and cooling. Yet, the assumption of a single effective electron temperature during the interaction of graphene with ultrafast electric fields describes the data very well in the field strength range up to 36 kV cm^−1^.

However, as the THz field strength increases from 36 kV cm^−1^ and up, the simple single-effective-temperature description used in Fig. 2c does not reproduce the data very accurately. This calls for a consideration of the time-dependent thermodynamic balance during the interaction of the electron gas in graphene with the ultrafast electric field, again using the statistical approach equation (1) to describe the instantaneous electron temperature *T*_el_. The electron gas heating and thermalization is again considered an instantaneous process on the THz timescale, while the electron gas cooling, which occurs via phonon emission, is considered a retarded, picosecond timescale effect with known dynamics 33,34,41,42, as described in the Supplementary Methods and shown in Supplementary Fig. 5. Adopting a split-step time-domain propagation method such as commonly used in nonlinear optics^43^ (see Methods for details), we simulate our THz nonlinear spectroscopy experiment (Fig. 1) and numerically propagate the THz field through the graphene sample, within the necessary constraints of conservation of carrier density *N*_s_=const, and conservation of energy by dynamically adding the energy of the absorbed THz field to and subtracting the energy corresponding to phonon emission from the total electron heat in graphene. The amount of energy transferred from the THz field to the electron gas in graphene can be directly inferred from the measured conductivity, as the real-valued conductivity of the medium is directly related to its power absorption coefficient. In this calculation only independently and experimentally determined quantities are used as inputs: the initial material parameters—the Fermi level *E*_F_ and the electron momentum scattering time of the sample *τ*(*E*_F_) determined at *T*_el_=300 K, the known electron cooling dynamics^33^,^34^ (see Supplementary Fig. 5) and the experimental incident THz waveforms *E*_THz_(*t*) in the entire range of peak field strength (see Fig. 1a). Electron momentum scattering is assumed to be dominated by Coulomb impurities, as concluded above (see Fig. 2c). The procedure provides the calculated THz conductivity spectra, which can be directly compared with the experimentally measured ones.

**Figure 3:**
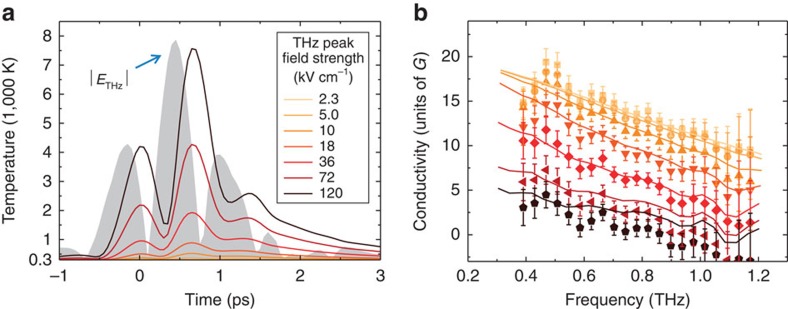
Dynamics of the carrier temperature in graphene, and nonlinear THz conductivity spectra for the full range of peak THz fields. (**a**) The calculated time-dependent carrier temperature in graphene during the interaction with the THz signal with peak fields in the range 2.3–120 kV cm^−1^. The absolute value of the experimentally measured ultrafast electric field transient, used as an input in the calculation, is shown as a grey area. Note the delayed, nonlinear response of the electron temperature to the THz field, reflecting the interplay between heating (near-instantaneous) and cooling (governed by the picosecond phonon cooling) dynamics of the electron gas. (**b**) The entirety of the measured conductivity spectra of graphene (symbols), along with the corresponding modelling (lines) based on time-dependent thermodynamic balance in graphene, for the full range of the peak THz fields used in experiments (colour scheme same as in **a**). The error bars are the s.d. in the measurements.

The time-domain calculation provides useful insights into the dynamical heat balance of the electron gas in graphene during the interaction with ultrafast electric fields. The temporal evolution of the instantaneous temperature *T*_el_ in graphene is shown in Fig. 3a, with the temporal shape of the (absolute value of the) applied THz electric field shown as a reference. As a consequence of the asymmetry in the dynamics of electron gas heating (instantaneous) and cooling (retarded)^33^,^34^, the heat is dynamically accumulated in the electronic population of graphene during the interaction with the electric field, and the electron temperature always exceeds the initial value of 300 K. The electron temperature follows the peaks in the driving electric field in a nonlinear and non-instantaneous manner. The complete cooling of the electron gas in graphene is achieved only several picoseconds after the main part of the electric signal has interacted with the material. One can see that the peak electron temperatures can reach (short-lived) values in excess of 7,000 K at the strongest peak electric field of 120 kV cm^−1^ (for this particular THz waveform used).

In Fig. 3b, as symbols, we show the complete set of measured conductivity spectra of our graphene sample, in the entire frequency and peak field strength ranges of our experiments, 0.4–1.2 THz and 2.3–120 kV cm^−1^, respectively. As solid lines in the same figure, we show the conductivity spectra calculated with the split-step time-domain method, corresponding to the same experimental conditions. Our modelling approach, not containing any free parameters and based on the simple time-dependent thermodynamic balance maintained in the electronic system of graphene, describes the entirety of our experimental observations very well (see also Supplementary Fig. 6). In particular, it reproduces the significant reduction of the graphene conductivity with the increase in both applied electric field strength and its oscillation frequency.

## Discussion

**Figure 4:**
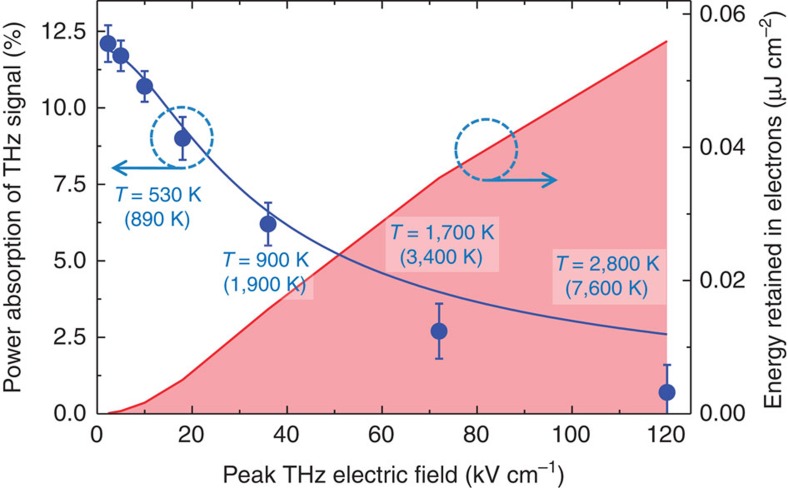
Power absorption of the ultrafast electric field signal, retained electronic heat and carrier temperatures. Measured (blue symbols), and predicted by the time-dependent thermodynamic model (blue solid line), integrated power absorption of graphene as function of peak THz field. Red area: the integrated energy retained in electronic system of graphene as a result of interaction with the THz field, provided by the model. The corresponding average and peak (in brackets) electron temperatures *T*_el_ for selected peak THz fields are indicated in the figure. The error bars are the s.d. in the measurements.

Our model allows us to quantify the efficiency of energy transfer from ultrafast electric fields to electron heat of graphene, shown (blue line) in Fig. 4 along with the experimentally determined (blue symbols) power absorption *A* of graphene as a function of peak THz electric field. The agreement between measurement and calculation lends further credence to our original supposition that the ultrafast conduction in graphene is determined simply by the thermodynamic balance maintained within its electron gas. For the selected values of THz field strengths in Fig. 4, we also show the peak (in brackets) and the time-averaged electronic temperatures in graphene, as produced by the model. The red line in Fig. 4 shows the calculated time-integrated amount of thermal energy *Q* retained within the electronic system of graphene over the course of interaction with the THz fields. We find that as much as *Q*≈0.06 μJ cm^−2^ of energy is retained as electronic heat in graphene in case of the strongest electric field applied, which is in fact comparable to the energies transferred to graphene in the experiments involving intense optical excitation^20^,^32^,^33^,^34^,^35^.

Using the values of the thermal energy retained in the electronic system *Q*, we calculate the average electron heating efficiency *η*=*Q*/(A × *F*_p_)—the ratio of the time-averaged thermal energy retained in the electron gas of graphene (a value produced by the calculation) to the total absorbed THz energy (an experimentally-measured value confirmed by the same calculation), where *F*_p_ is the known fluence of the THz signal, see Supplementary Fig. 7. We find that over the timescale of interaction with our 1.5–2.0 ps-long electric field pulses, the heating efficiency is maintained at about *η*≈15% for the whole range of peak THz fields (2.3–120 kV cm^−1^), with the rest of the deposited electric field energy being converted to phonons on the same timescale. We emphasize that these values are specific not only to the cooling dynamics of the graphene sample, but also to the actual shape of the THz waveform, defining the dynamics of the highly nonlinear interaction between the electric field and graphene (see Fig. 3a).

The high electron temperatures we find (see Fig. 3a and Fig. 4) imply that the high-energy tail of the hot-carrier distribution (see Fig. 2a,b) will extend to energies well above the Fermi level of graphene at 300 K, *E*_F_=0.07 eV. The population-averaged electron energy as a result of the strongest electric field excitation (120 kV cm^−1^ field strength) yields the value *Q*/*N*_s_∼0.5 eV. Indeed, electrons with energies as high as 0.78 eV were observed by optical probing of the density of states of doped graphene, excited with ultrafast THz fields of ∼200 kV cm^−1^ strength in ref. 44. We note that in this regime, with electrons reaching energies exceeding the optical phonon energy in graphene of 0.2 eV (see Supplementary Fig. 8), electron cooling leads to an increase of the optical phonon population. This can result in a reduction of the conductivity by adding an additional momentum scattering channel: electron–phonon scattering^20^,^39^,^45^,^46^ (see also Supplementary Note 1). This would also lead to a deviation from the linear relation between scattering time and carrier energy, especially for high-energy carriers, and could explain the small discrepancy between the data and our simple model at the highest field strengths in Fig. 4 (see also Supplementary Note 1). Here the measured absorbance of graphene is even lower than predicted, indicating that additional scattering channels, likely involving phonons, start contributing. Finally, we note that all microscopic electron kinetic processes involved in the transfer of energy from ultrafast electric fields to the electronic system of graphene (see for example, refs 47, 48, 49, 50) are implicitly included in our simple thermodynamic approach, which relies on the fact that the energetic electrons in graphene thermalize much faster than the THz field oscillates.

In conclusion, we have shown that the ultrafast electron transport in single-layer graphene can be understood within a simple picture of a time-dependent thermodynamic (that is, statistically determined) balance maintained within the electronic population of graphene: The primary mechanism of interaction between the electric fields and graphene is the conductivity, directly converting the energy of the applied electric field into the energy of induced electric current. However, the extremely efficient electron–electron interaction in graphene^30^,^31^,^32^,^33^,^34^ near-instantaneously converts the energy of electric current into thermal energy of the entire electron gas in graphene. This leads to an elevation of the common electronic temperature, smearing-out of the Fermi–Dirac distribution and a concomitant lowering of the chemical potential of the electron gas. This, in turn, leads to the reduced intra-band, THz-frequency conductivity.

The timescale for electron gas thermalization in graphene, previously determined to be sub-50 fs (refs 30, 33, 34), naturally limits the timescale on which our simple thermodynamic picture of electron transport is applicable. At the same time, the (sub-)picosecond timescale of charge transport in graphene, investigated in this work, definitely covers the regime that is directly relevant to several applications in the promising field of ultrafast graphene electronics. As graphene shows reduced conductivity in response to both stronger electric fields and higher frequencies as a result of electron gas heating, this may become important for ultrafast graphene transistors with channel fields on the order of 100 kV cm^−1^, operating at ∼THz frequencies^9^,^10^,^51^. However, the reduction of conductivity in graphene under such a strong-field, ultrafast modulation may be mitigated by, for example, using a material with a higher doping level, resulting in a smaller increase in electron temperature as the energy of the electric current will be shared by a larger number of carriers in the electron gas. Since the dynamic heat accumulation in graphene on the THz timescale is the primary cause of the high-frequency conductivity reduction, the ultrafast conductivity of graphene can be also enhanced by providing faster electron cooling channels, for example, by substrate engineering, leading to increased coupling of electrons in graphene to substrate phonons.

On the other hand, the high electron temperatures and relatively slow cooling dynamics in graphene are clearly advantageous for THz detectors^15^,^16^ based on the extraction of high-energy electrons into an external circuit^52^, or on the ultrafast modulation of optical signals with the THz signals^53^. The detector efficiency could be enhanced, for example, by lower initial doping producing higher effective electron temperatures, and by engineered reduced electron–phonon coupling. Further, as the electron momentum scattering rate depends on the electron energy^4^,^40^, this can have implications for THz plasmonic devices^17^,^18^, where the plasma damping can be controlled in this way. Finally, the strong optical nonlinearity of graphene in the THz range, controlled by the doping level, can be directly employed in THz modulators^19^ operating in the nonlinear regime^25^,^54^.

## Methods

### Split-step time-domain calculation of nonlinear conductivity spectra

We propagate the incident THz pulse numerically through the sample, thus calculating the transmitted waveform *E*_trans_(*t*) (through the substrate with graphene), from the known, measured reference waveform *E*_ref_(*t*) (through the substrate without graphene). As the sample response and the field strength in the THz waveform are coupled to each other through the absorbed heat *Q*(*t*) of the carriers, we numerically solve the following system of nonlinear equations. The transmitted waveform is the convolution of the response function of the sample and the incident waveform:


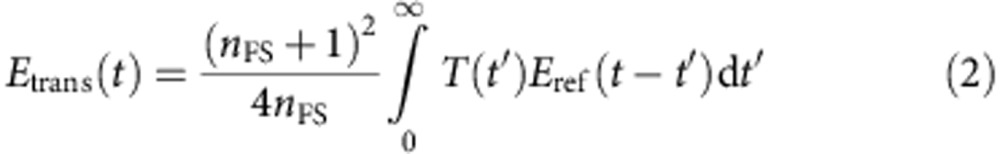


where *T*(*t*) is the inverse Fourier transform of the complex transmission function *t*(*ω*) (see Supplementary Methods). Note that this parameter depends on the heat absorbed by the carriers from the THz pulse *Q*(*t*). We calculate this from the energy balance of the experiment—the absorbed THz energy is equal to the difference between the energy of the incident THz waveform and that of the reflected and transmitted waveforms:


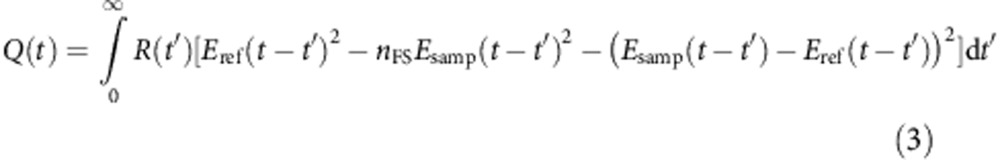


where *R*(*t*) describes the dynamics of the heat dissipation from the carriers. We take these dynamics from the experimental study in ref. 33 (see also Supplementary Methods). From the coupled equations (2) and (3), we numerically calculate *E*_trans_(*t*). Finally we obtain the nonlinear conductivity using equation (1).

## Additional information

**How to cite this article:** Mics, Z. *et al*. Thermodynamic picture of ultrafast charge transport in graphene. *Nat. Commun.* 6:7655 doi: 10.1038/ncomms8655 (2015).

## Supplementary Material

Supplementary Information Supplementary Figures 1-8, Supplementary Note 1, Supplementary Methods and Supplementary References
