# Supplementary material for: Thermodynamic picture of ultrafast charge transport in graphene
Source: Nat Commun. 2015 Jul 16;6:7655. doi: 10.1038/ncomms8655 (PMC4518297; doi:10.1038/ncomms8655)
Supplement: Supplementary Information — Supplementary Figures 1-8, Supplementary Note 1, Supplementary Methods and Supplementary References [file ncomms8655-s1.pdf]

SUPPLEMENTARY FIGURES

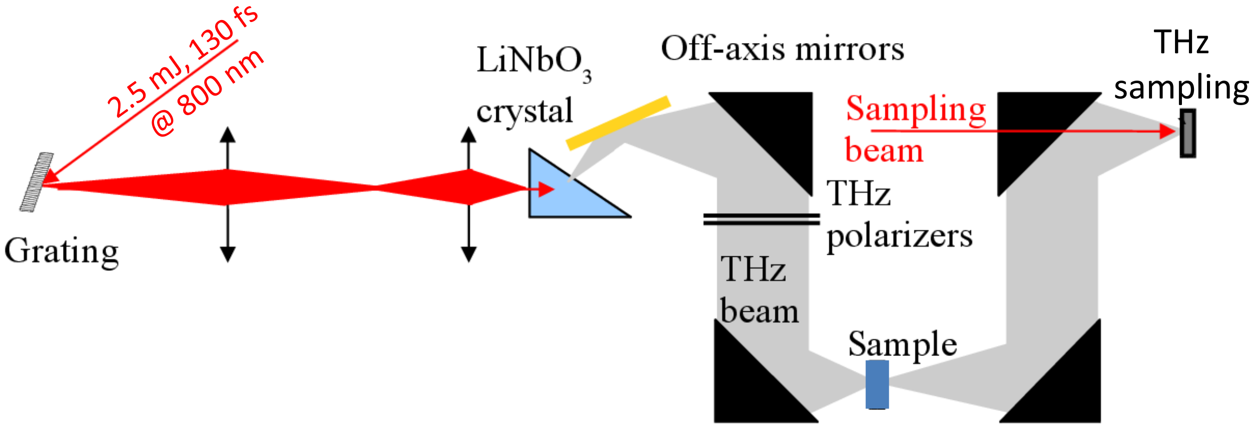

Supplementary Figure 1. Schematic representation of the high-field THz spectroscopy setup.

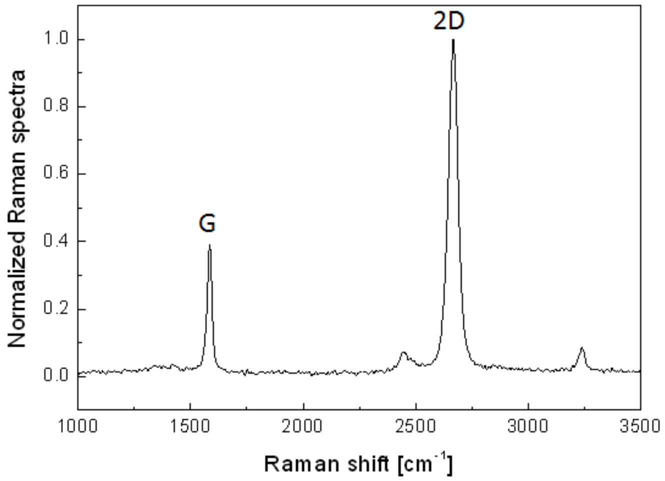

Supplementary Figure 2. The measured Raman spectrum (with 532 nm pump) of the graphene sample. The Gaussian nature of the 2D line shows that our sample is single layer graphene. The position of the G peak is ~1586 cm^{-1}, and the ratio between the 2D and the G peak is ~2.6. From this we conclude that the Fermi-level is <0.14 eV [1,2]. The negligible D line intensity (at ~1350 cm^{-1}) indicates high quality CVD graphene.

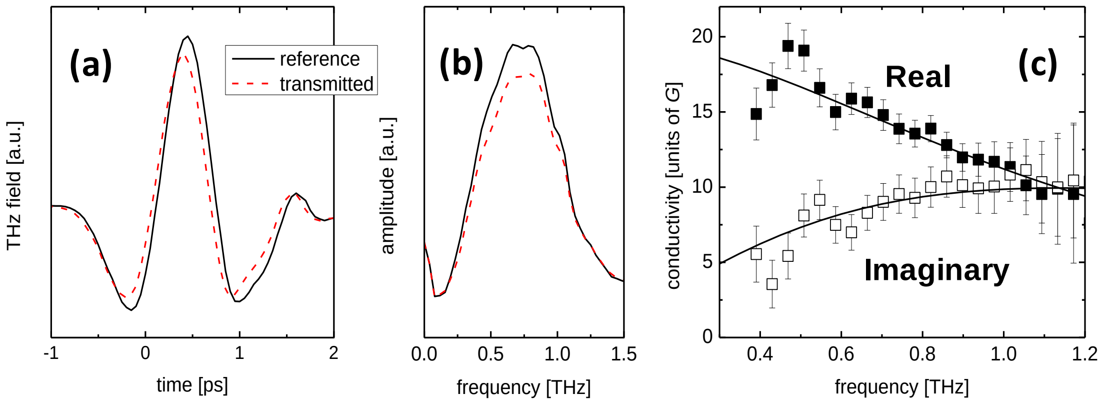

Supplementary Figure 3. (a) Comparison of the field profiles of THz pulses transmitted through a bare fused silica substrate ('reference') and through our sample that contains CVD graphene deposited on a fused silica substrate ('transmitted'). **(b)** Comparison of the pulses in the frequency domain. **(c)** Extracted *linear* THz conductivity of the graphene sample, normalized by $G_0 = e^2/4\hbar$ at low peak THz fields (< 2kV cm^{-1}), with solid lines representing the fit result using $\sigma_{int}(\omega) = \frac{e_0^2}{\pi\hbar^2}|E_F|\frac{\tau}{1-i\ \omega\tau}$ [3]. As fit parameters we obtain a momentum scattering time of $\tau = (140 \pm 20)$ fs, and a Fermi level of $E_F = (0.07 \pm 0.01)$ eV, in agreement with the Raman spectrum. Similar values for the scattering time in similar samples were reported in previous studies [3,4].

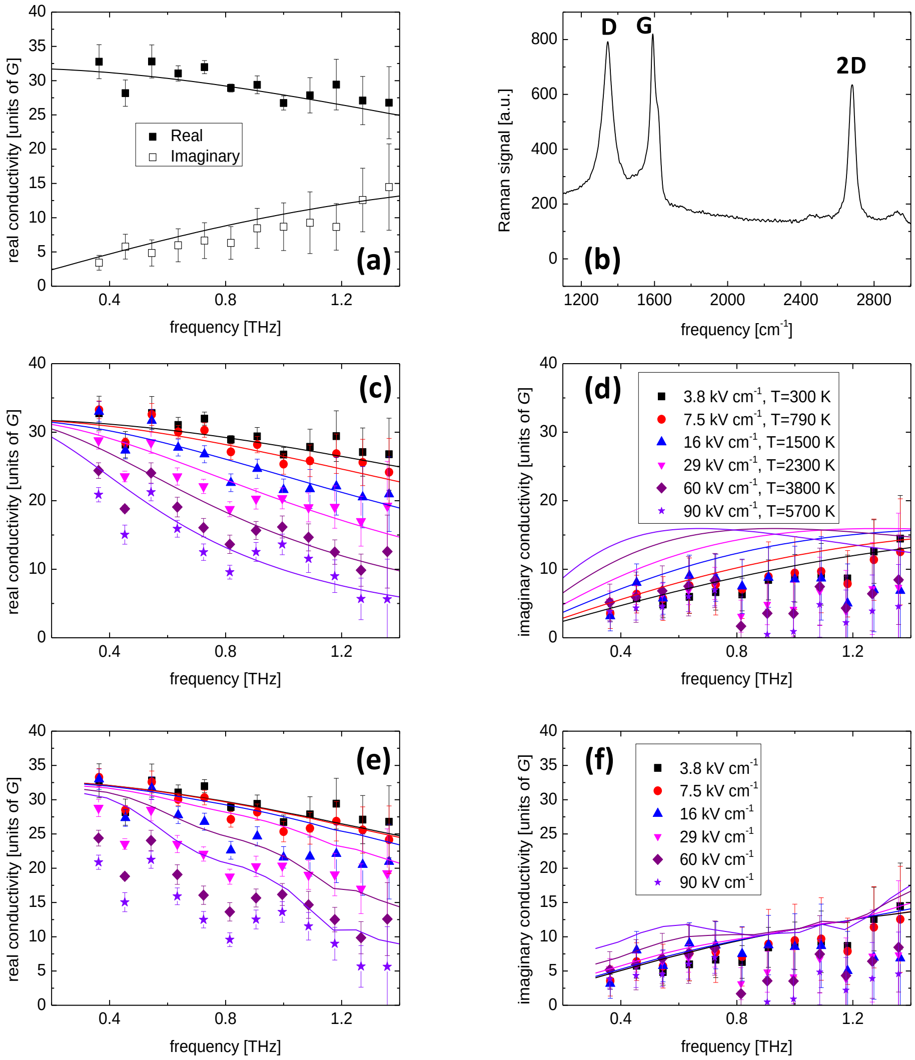

Supplementary Figure 4. (a) Linear THz characterization, **(b)** Raman spectrum, and **(c,d)** real and imaginary nonlinear THz conductivity spectra for the alternative CVD graphene sample with E_F = 230 ± 40 meV, N_s = (8.0 ± 2.0) ×10^{12} cm^{-2}, and $\tau(E_F)$ = 60 ± 20 fs, with solid lines representing fits using Eq. 1 in the main text. **(e,f)** The measured nonlinear conductivity spectra, now with solid lines representing the split-step time-domain model.

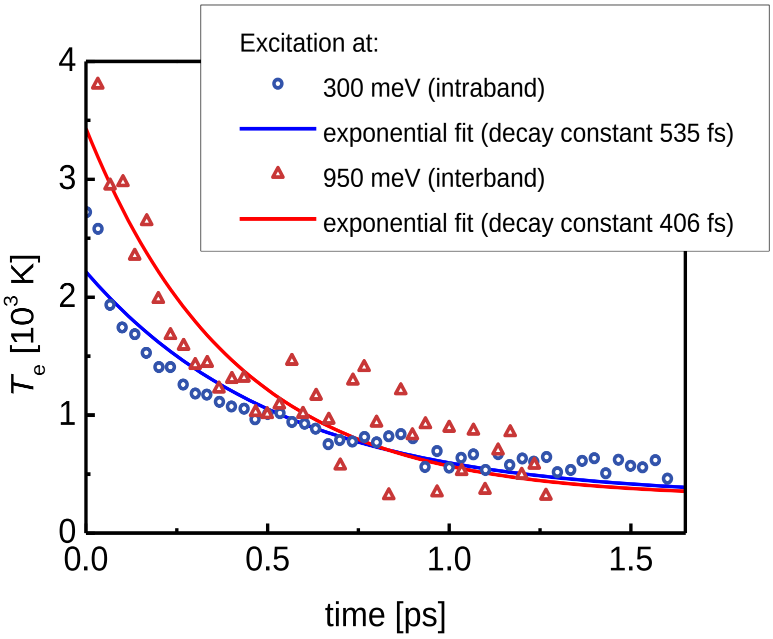

Supplementary Figure 5. Electron cooling dynamics in single-layer doped graphene for the case of intraband excitation (300 meV, blue circles) and inter-band excitation (950 meV, red triangles), adopted from the fs ARPES measurements in Ref. [5]. The lines represent single exponential fits to the data with very close time constants, indicating the robustness of electron cooling dynamics in graphene towards excitation conditions.

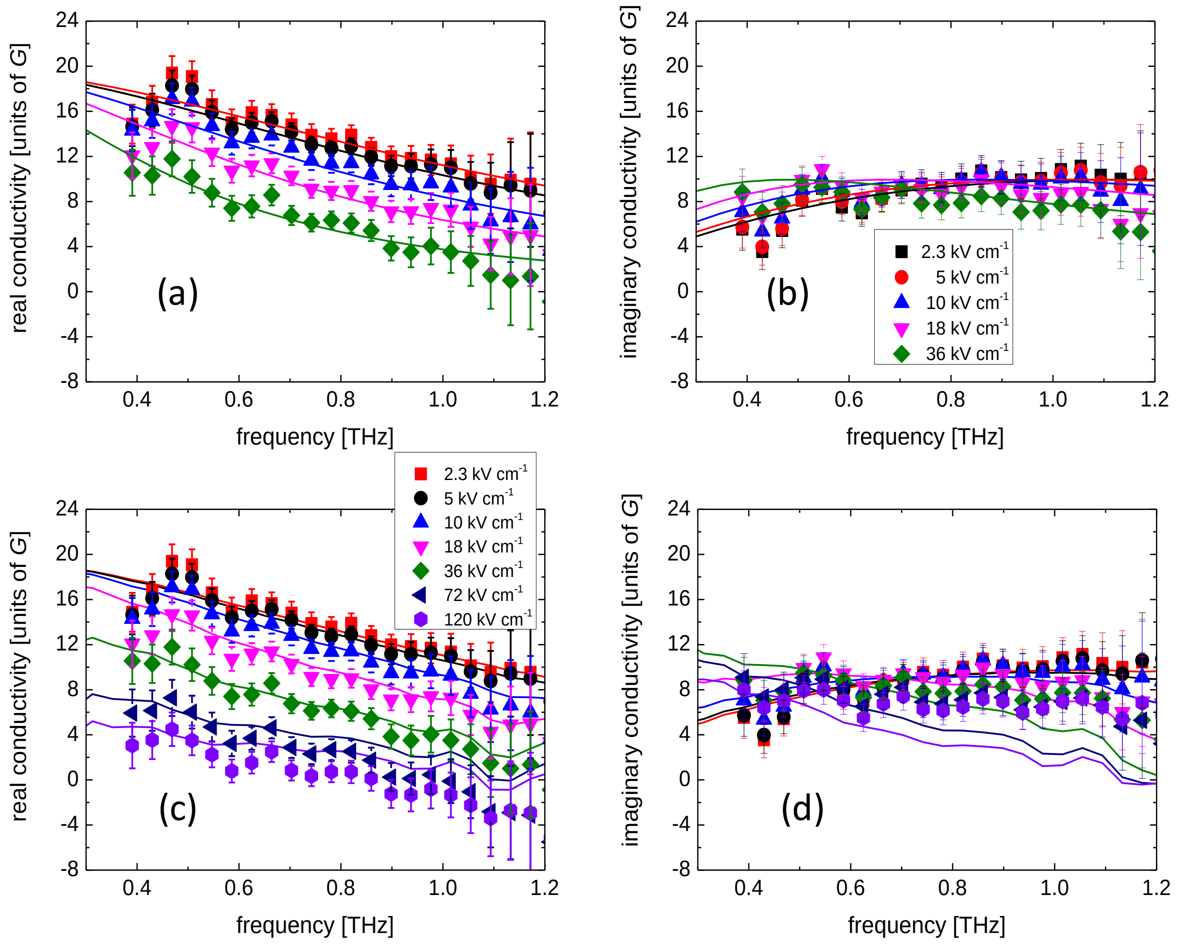

Supplementary Figure 6. The measured conductivity spectra, with solid lines representing **(a,b)** fits to Eq. 1 in the main text or **(c,d)** representing the spectra calculated using the split-step time-domain model.

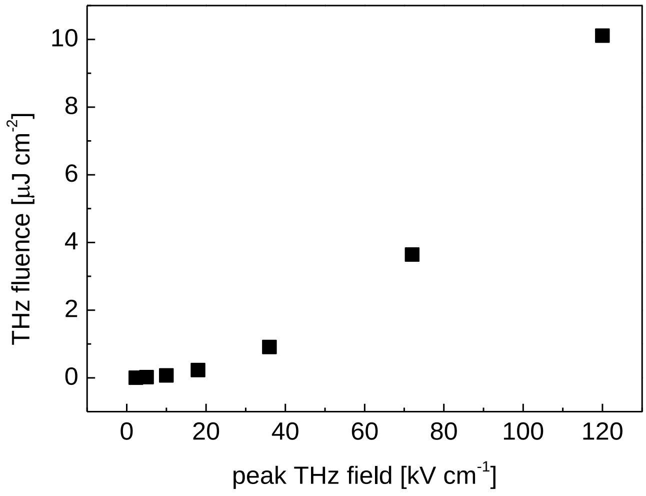

Supplementary Figure 7. THz energy fluence versus the measured peak THz field.

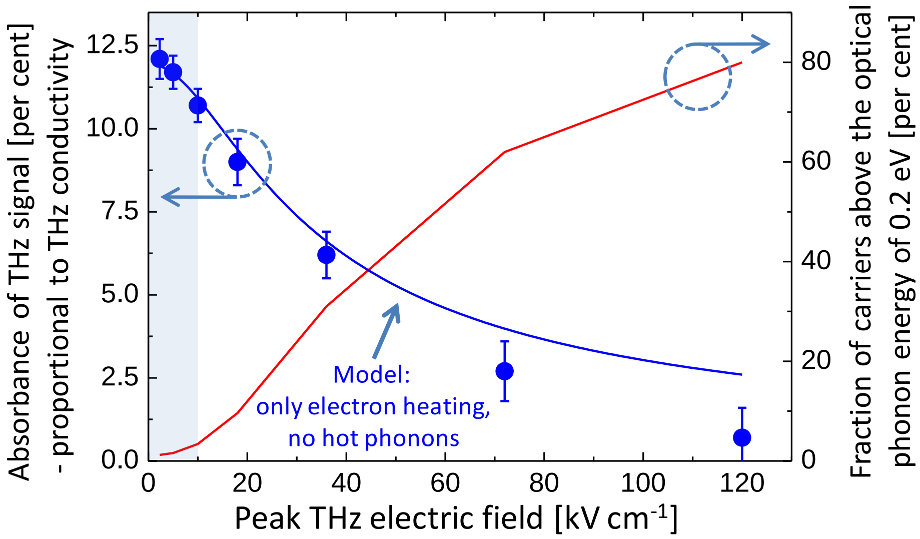

Supplementary Figure 8. Total THz absorbance (left vertical axis) and the fraction of carriers with excess energy above the optical phonon energy (right vertical axis) as a function of peak THz field. The figure, in particular the shaded area, shows that the THz absorption decreases significantly (>20 %), even when the fraction of carriers that can emit an optical phonon is low (<10 %).

Supplementary Note 1

The effect of hot phonon scattering

Here we estimate the influence of electron scattering on hot phonons, in THz transport in graphene, within the framework of available experimental data. In the regime of hot phonon scattering, the increase of the optical phonon population (if absorbed electrical energy is transferred to the phonon system) in graphene can lead to an increased electron-phonon scattering efficiency, and hence to an overall reduction of the electron mobility in graphene. The probability of such a scenario in our experiments can be estimated using the basic arguments of energy and particle number conservation. Let us consider an extreme-case scenario, when all the absorbed electric field energy (which is a directly measured quantity in our experiment) is transferred to electronic heat, extending the tail of the Fermi-Dirac distribution of electron gas to high energies. Only the electrons with energy higher than the optical phonon energy of 0.2 eV can emit an optical phonon. The fraction of electrons with an energy exceeding the optical phonon energy is readily calculated based on the known carrier density N_s and the thermal energy of electron gas as $F_{ph} = \frac{\int_{0.2eV}^{\infty} D(E) f_{FD}(E,T) dE}{N_S}$. Its effect should now be compared with the observed reduction in the THz absorption of graphene (see Supplementary Figure 8), which is a direct measure of the reduction in graphene conductivity. At a weak electric field strength of 5 kV cm^{-1}, only F_{ph} =1.2% of all electrons are able to emit an optical phonon, yet the conductivity of graphene is already reduced by about 3.5% with respect to its maximum measured in the non-perturbative regime at the weakest electric field of 2.3 kV cm^{-1}. At somewhat stronger electric field of 10 kV cm^{-1}, when only F_{ph} = 3.4% of all electrons can emit a phonon, the conductivity of graphene is already reduced by a very significant 12% with respect to its maximum. This shows a significant disproportion between the increasing phonon population and the observed reduction of graphene conductivity.

Further, at these weaker applied electric fields, the electron temperature in graphene remains well below the Fermi temperature $T_F = E_F/k_B$ = 812 K (see Fig. 2(c) in the main text). In [6] it was shown that under such conditions, and in the case of low absorbed thermal energy < 0.1 μJ cm^{-2}, the phonon emission effect on the electron mobility will be negligible even if the entire electron population would contribute to the generation of optical phonons. This is obviously the case for graphene with a mobility that is not limited by phonon scattering, but by impurity scattering, as is the case in our sample. Therefore, based on the above arguments we conclude that the effect of hot phonons on the observed reduction of THz conductivity of graphene is not substantial, at least in the case of weak and moderate carrier temperatures $T_{el} \leq T_F$ where most of the conductivity reduction in graphene is already achieved (see Figs. 2(c) main text and Supplementary Figure 8). Yet, the hot phonon contribution will increase in significance for the highest electronic temperatures, where it may explain the small discrepancy between the measured graphene absorption, and the one modeled using exclusively the thermal balance in the electron system (see Supplementary Figure 6 (c,d)).

SUPPLEMENTARY METHODS

Nonlinear THz spectroscopy setup

The high-field THz spectroscopy setup is driven by ultrafast laser pulses with a central wavelength of 800 nm, a pulse duration of 130 fs and a pulse energy of 2.5 mJ. The wavefront of the laser pulses is tilted using a strong 2000 mm^{-1} grating, and the diffracted laser beam is imaged by a system of cylindrical lenses onto a $LiNbO_3$ crystal (see Supplementary Figure 1). By non-collinear optical rectification of the incident laser beam in the $LiNbO_3$ crystal, intense THz pulses with pulse energy up to 1.4 μJ are generated [7]. The THz pulses are collimated and focused on the sample by a pair of parabolic mirrors with focal lengths of 7'' and 3'', respectively. The strength of the THz field incident on the sample is controlled by a pair of wire-grid polarizers. The THz beam transmitted through the sample is collimated and focused on a (110)-oriented ZnTe crystal. In the ZnTe crystal the THz beam overlaps with a weak 800 nm sampling beam. In the ZnTe crystal, the polarization of this sampling beam is changed upon the presence of the electric field of the overlapping THz pulse. By changing the delay between the 800 nm sampling pulse and the THz pulse, the field profile of the THz pulse is measured (electrooptic sampling). Saturation of the detector at high THz fields is prevented by inserting 1 mm thick silicon plates into the collimated THz beam between the sample and the detector; each of the plates attenuates the THz beam by Fresnel losses at the interfaces by 70%, without causing any dispersion to the THz signal. The THz beam path in the spectrometer is purged with dry nitrogen to avoid the effects of THz absorption by atmospheric water. All the measurements in this work are carried out at room temperature.

THz data analysis

First we discuss how we extract the THz sheet conductivity. We specifically emphasize here that the conductivity σ measured in the time domain, is the directly measured proportionality between the driving electric field and the driven current $j = \sigma E_{THz}$. Immediately reflecting the conductive response of the material to the driving field, it can naturally be field-dependent in case of nonlinear material response. As such, it should not be confused with the conductivity narrowly defined within a linear, field-strength independent concept.

Due to the intrinsic doping of graphene on fused sililca, the intrinsic conduction band carriers lead to absorption of an incident terahertz pulse. Consequently, the THz transmission through a graphene layer on a fused silica substrate is lower than that through a bare fused silica substrate. We measure the time-domain electric field profile of THz pulses transmitted through the sample $E_{trans}(t)$ (i.e. graphene on fused silica) and that of THz pulses transmitted through a bare fused silica substrate $E_{ref}(t)$ (see Supplementary Figure 3 (a,b)). After applying Fourier transform to the THz field waveforms, we obtain the sheet THz conductivity spectra of the graphene layer directly using the Tinkham equation [8]– a solution of Maxwell's equations, which is valid for conductive thin films:

$$\sigma_{sheet}(\omega) = \frac{n_{FS}+1}{Z_0}\left(\frac{E_{ref}(\omega)}{E_{trans}(\omega)} e^{\frac{i\Delta d\omega}{c}} - 1\right) \quad (1)$$

where n_{FS} = 1.95 is the index of refraction of the substrate and Z_0 = 377 Ω is the impedance of free space. Δd is the difference between the thickness of the reference sample without graphene and the sample with graphene. We measure this difference in thickness using an optical autocorrelator: first we record the autocorrelation function for the reference sample without graphene and then for the sample with graphene. The difference between the two autocorrelation functions gives us the thickness difference Δd. The thickness difference corresponds to the difference in thickness between the two different substrates and in our case is 11±1 μm.

Next, we discuss how we extract the nonlinear conductivity. For this, we compare the THz pulses incident onto the sample and the THz pulses transmitted through the sample in the time domain. After Fourier transformation, we obtain the complex-valued THz-frequency field transmission function $\hat{T}(\omega)$, which is directly related to the complex-valued THz (sheet) conductivity spectrum of graphene $\hat{\sigma}(\omega)$ via the Tinkham equation [8] $\hat{T} = \frac{n+1}{n_{FS}+1+Z_0\hat{\sigma}}$.

The values of the field strength of the THz pulses incident onto the sample are extracted as follows. For this , we use electrooptic sampling in a 0.2 mm thick (110)-oriented ZnTe crystal that is placed at the position of the sample. The THz field strength is then directly measured from the change of the ellipticity of a weak sampling laser pulse propagating through the ZnTe crystal simultaneously with the THz pulse [9]. We calculate the power irradiation of the sample by the THz beam by integrating the square of the electric field temporally (see Supplementary Figure 7). We estimate a relative error of 20% in determining the fluence of the THz beam. The field strength of the THz pulses represents the peak electric field at the center of the THz beam at the position of the sample (the focal point of the 2nd parabolic mirror). In the experiment we monitor the THz transmission only at the peak (center) of the THz pulse. We ensure this by focusing the 800 nm sampling beam into the center of the THz focus after the 4th parabolic mirror.

Finally, we discuss how we extract the power absorption of the graphene layer. From the time-domain transmission measurement we directly determine the power absorption of the graphene layer. By taking into account that the graphene film is very thin and conductive, the transmission function of the graphene layer is [8]:

$$t(\omega) = \frac{2}{n_{FS}+1+Z_0\sigma_{sheet}(\omega)} \quad (2)$$

Note that the THz pulse transmitted through a graphene layer propagates through the substrate, an optical medium with refracting index n_{FS}. For the reflection coefficient we find:

$$r(\omega) = t(\omega) - 1 \quad (3)$$

This identity allows us to determine the power absorption coefficient of the sample directly from the measured temporal profiles of the THz pulses:

$$A = 1 - \frac{n_{FS} \int E_{trans}(t)^2 dt + \int (E_{trans}(t) - E_{ref}(t))^2 dt}{\int E_{ref}(t)^2 dt} \quad (4)$$

Here the integration is carried out over the whole temporal profile of the THz pulses, and we use the fact that the intensity of light is proportional to the index of refraction of the medium and the square of the electric field.

Sample growth

Due to the long wavelength of THz radiation, the focus spot in our experiment is on the order of 1 mm. Since our setup thus requires large area graphene samples, we use graphene grown by chemical vapor deposition (CVD). The graphene film was grown on 25 µm thick Cu foils (Alfa Aesar, item No.13382) in a hot wall furnace consisting of a 50 mm inner diameter quartz tube and heated in a split tube furnace. The quartz tube was loaded with the Cu foil. The tube was then evacuated down below 5×10^{-3} mTorr. The pressure was maintained at ~1 mTorr under 100 sccm H_2 flow. The tube was heated up to 1020 °C (heating ramp rate 25 °C per min) and the Cu foil was annealed at for 40 min. The annealing was followed by introducing 20 sccm of CH_4 for 20 min. After the exposure to CH_4, the furnace was cooled to room temperature. The CH_4 was turned off when the temperature was below 600 ^{o}C and H_2 was turned off when the temperature was below 200 ^{o}C.

The graphene layer was subsequently transferred from the copper foil onto a 1 mm thick fused silica substrate using the following procedure. Polymethyl methacrylate (PMMA, product number: 950 PMMA C2 from MicroChem) solution was spin coated at 2000 r.p.m for 60s on the top surface of the graphene film on the Cu foil (which was fixed on a Si wafer) and annealed at 80 °C for 2 min to achieve the cure of the polymer. The Cu foil was then etched overnight using aqueous $Fe(NO_3)_3$ (Sigma-Aldrich) (0.1 g per mL) solution . The floated graphene on PMMA support was transferred to a deionized water (DI) bath, rinsed several times with DI water and isopropanol to fully wash off residual Cu etchant. The graphene film was transferred onto the target fused silica (1 cm × 1 cm) substrate. Afterwards, the PMMA/graphene/quartz film was exposed to acetone vapor at 75 °C for 30 min to remove the PMMA layer. Finally, the samples were rinsed in isopropanol several times and dried under nitrogen flow.

Hot electron cooling dynamics

We extract the relaxation rate for hot electrons directly from the experimental results in Ref. [5]. Here, the carrier cooling dynamics after both inter- and intra-band excitations of graphene were measured and quantified using fs ARPES [5]. Their graphene sample is similar to the one we use. The data in this work are perfectly consistent with an independently performed experiment by a different group [10]. The electron cooling dynamics in [5], measured in the cases of intra-band (pump energy 300 meV) and inter-band (pump energy 950 meV), are reproduced in Supplementary Figure 5.

As it is evident from Supplementary Figure 5, the difference between the electron cooling dynamics for inter- and intra-band excitations of graphene, corresponding to entirely different excitation conditions, is relatively minor. In view of this similarity in the cooling dynamics, measured under the excitation when the

photon energies differ by a factor of more than 3, and under the difference excitation fluence, it is reasonably accurate to use the intra-band relaxation dynamics found in [5] in our calculation.

Based on the carrier cooling characterized in [5], the functional form we use for the dissipation of excess heat from the carrier system is:

$$R(t) = 0.54 \exp\left(-\frac{t}{0.1}\right) + 0.46 \exp(-\frac{t}{0.92}) \quad (5)$$

Here t is time in picoseconds. We note that these cooling dynamics likely correspond to a combination of hot electrons coupling to graphene optical phonons (the sub-picosecond component) and impurity-assisted supercollision cooling to acoustic graphene phonons [11–13], and/or energy flow to the substrate [14] (the picosecond component).

Supplementary references

1. H. Yan, F. Xia, W. Zhu, M. Freitag, C. Dimitrakopoulos, A. A. Bol, G. Tulevski, and P. Avouris, "Infrared spectroscopy of wafer-scale graphene," ACS Nano **5**, 9854–9860 (2011).

2. A. Das, S. Pisana, B. Chakraborty, S. Piscanec, S. K. Saha, U. V Waghmare, K. S. Novoselov, H. R. Krishnamurthy, A. K. Geim, A. C. Ferrari, and A. K. Sood, "Monitoring dopants by Raman scattering in an electrochemically top-gated graphene transistor," Nat. Nanotechnol. **3**, 210–215 (2008).

3. G. Jnawali, Y. Rao, H. Yan, and T. F. Heinz, "Observation of a transient decrease in terahertz conductivity of single-layer graphene induced by ultrafast optical excitation," Nano Lett. **13**, 524–530 (2013).

4. H. Y. Hwang, N. C. Brandt, H. Farhat, A. L. Hsu, J. Kong, and K. A. Nelson, "Nonlinear THz conductivity dynamics in P-type CVD-grown graphene," J. Phys. Chem. B **117**, 15819–15824 (2013).

5. I. Gierz, J. C. Petersen, M. Mitrano, C. Cacho, I. C. E. Turcu, E. Springate, A. Stöhr, A. Köhler, U. Starke, and A. Cavalleri, "Snapshots of non-equilibrium Dirac carrier distributions in graphene," Nat. Mater. **12**, 1119–1124 (2013).

6. K. J. Tielrooij, J. C. W. Song, S. A. Jensen, A. Centeno, A. Pesquera, A. Zurutuza Elorza, M. Bonn, L. S. Levitov, and F. H. L. Koppens, "Photoexcitation cascade and multiple hot-carrier generation in graphene," Nat. Phys. **9**, 248–252 (2013).

7. K.-L. Yeh, M. C. Hoffmann, J. Hebling, and K. A. Nelson, "Generation of 10 μJ ultrashort terahertz pulses by optical rectification," Appl. Phys. Lett. **90**, 171121 (2007).

8. R. E. Glover and M. Tinkham, "Conductivity of superconducting films for photon energies between 0.3 and 40kT_c," Phys. Rev. **108**, 243–256 (1957).

9. P. C. M. Planken, H.-K. Nienhuys, H. J. Bakker, and T. Wenckebach, "Measurement and calculation of the orientation dependence of terahertz pulse detection in ZnTe," J. Opt. Soc. Am. B **18**, 313–317 (2001).

10. J. C. Johannsen, S. Ulstrup, F. Cilento, A. Crepaldi, M. Zacchigna, C. Cacho, I. C. E. Turcu, E. Springate, F. Fromm, C. Raidel, T. Seyller, F. Parmigiani, M. Grioni, and P. Hofmann, "Direct view of hot carrier dynamics in graphene," Phys. Rev. Lett. **111**, 027403 (2013).

11. J. C. W. Song, M. Y. Reizer, and L. S. Levitov, "Disorder-assisted electron-phonon scattering and cooling pathways in graphene," Phys. Rev. Lett. **109**, 106602 (2012).

12. A. C. Betz, S. H. Jhang, E. Pallecchi, R. Ferreira, G. Fève, J.-M. Berroir, and B. Plaçais, "Supercollision cooling in undoped graphene," Nat. Phys. **9**, 109–112 (2012).

13. M. W. Graham, S.-F. Shi, D. C. Ralph, J. Park, and P. L. McEuen, "Photocurrent measurements of supercollision cooling in graphene," Nat. Phys. **9**, 103–108 (2012).

14. M. Freitag, T. Low, and P. Avouris, "Increased responsivity of suspended graphene photodetectors," Nano Lett. **13**, 1644–1648 (2013).
